# Cardiology Encounters for Underrepresented Racial and Ethnic Groups with Human Immunodeficiency Virus and Borderline Cardiovascular Disease Risk

**DOI:** 10.1007/s40615-023-01627-0

**Published:** 2023-05-09

**Authors:** Gerald S. Bloomfield, C. Larry Hill, Karen Chiswell, Linda Cooper, Shamea Gray, Chris T. Longenecker, Darcy Louzao, Keith Marsolo, Eric G. Meissner, Caryn G. Morse, Charles Muiruri, Kevin L. Thomas, Eric J. Velazquez, Joseph Vicini, April C. Pettit, Gretchen Sanders, Nwora Lance Okeke

**Affiliations:** 1 Department of Medicine, Duke University School of Medicine, Durham, NC USA; 2 Duke Clinical Research Institute, Duke University School of Medicine, 300 W. Morgan Street, Durham, NC 27701 USA; 3 Department of Medicine, Medical University of South Carolina, Charleston, SC USA; 4 Division of Cardiology and Department of Global Health, University of Washington, Seattle, WA USA; 5 Department of Population Health Sciences, Duke University School of Medicine, Durham, NC USA; 6 Wake Forest University Health Sciences, Winston-Salem, NC USA; 7 Section of Cardiovascular Medicine, Yale University, New Haven, CT USA; 8 Department of Medicine, Vanderbilt University, Nashville, TN USA

**Keywords:** HIV, Cardiovascular disease, Underrepresented racial and ethnic groups, Referral and consultation, Risk factors

## Abstract

**Background:**

Underrepresented racial and ethnic groups (UREGs) with HIV have a higher risk of cardiovascular disease (CVD) compared with the general population. Referral to a cardiovascular specialist improves CVD risk factor management in high-risk individuals. However, patient and provider factors impacting the likelihood of UREGs with HIV to have an encounter with a cardiologist are unknown.

**Methods:**

We evaluated a cohort of UREGs with HIV and borderline CVD risk (10-year risk ≥ 5% by the pooled cohort equations or ≥ 7.5% by Framingham risk score). Participants received HIV-related care from 2014–2020 at four academic medical centers in the United States (U.S.). Adjusted Cox proportional hazards regression was used to estimate the association of patient and provider characteristics with time to first ambulatory cardiology encounter.

**Results:**

A total of 2,039 people with HIV (PWH) and borderline CVD risk were identified. The median age was 45 years (IQR: 36–50); 52% were female; and 94% were Black. Of these participants, 283 (14%) had an ambulatory visit with a cardiologist (17% of women vs. 11% of men, p < .001). In fully adjusted models, older age, higher body mass index (BMI), atrial fibrillation, multimorbidity, urban residence, and no recent insurance were associated with a greater likelihood of an encounter with a cardiologist.

**Conclusion:**

In UREGs with HIV and borderline CVD risk, the strongest determinants of a cardiology encounter were diagnosed CVD, insurance type, and urban residence. Future research is needed to determine the extent to which these encounters impact CVD care practices and outcomes in this population.

**Trial Registration:**

ClinicalTrials.gov Identifier: NCT04025125.

**Supplementary Information:**

The online version contains supplementary material available at 10.1007/s40615-023-01627-0.

## Introduction

People with HIV (PWH) are at an increased risk for major adverse cardiovascular events, including myocardial infarction, heart failure, stroke, and sudden cardiac death [1–3]. In addition to biologic mechanisms, such as HIV-related chronic inflammation and antiretroviral-associated metabolic disorders, healthcare system, patient, and clinician factors are also implicated in the greater burden of cardiovascular disease (CVD) among PWH [4, 5]. These factors include, but are not limited to, differences in access to specialty care, treatment differences, and the lack of clear clinical guidelines for CVD risk management, which have all been associated with worse CVD care and outcomes among PWH compared with the general population [6–8]. This burden is particularly acute in the Southern United States (U.S.), a region that accounts for more than half of all incident HIV infections [9]. Underrepresented racial and ethnic groups (UREGs) and women with HIV in the Southern U.S. are disproportionately affected as these groups have a greater burden of CVD risk factors and poorer HIV and CVD outcomes[10–12].

Despite awareness of CVD prevention and risk reduction in PWH as national research and implementation priorities, there remains significant heterogeneity in how CVD risk factors are managed in PWH. These differences in CVD risk factor management are partially accounted for by clinician specialty and heterogeneity in the likelihood of HIV clinicians to refer patients to cardiology specialist care [13, 14]. For example, PWH receiving primary care services from their HIV provider are less likely to be prescribed guideline-indicated statin therapy than patients in an internal medicine clinic [15]. These challenges are compounded by sex-based differences in access to specialty care [16, 17]. Inclusion of a cardiovascular specialist in the care team for high-risk individuals has been shown to improve CVD risk factor management in the general population [18]. Unfortunately, UREG individuals and women are less likely to be referred to cardiologists compared with White patients and men, even those at high risk for CVD [19, 20].

We currently lack sufficient knowledge of the factors that impact cardiology specialist care for UREG PWH, a population disproportionately impacted by elevated CVD risk and poor CVD outcomes. This knowledge is crucial to develop evidence-based strategies to provide optimal and equitable CVD care for all PWH. Therefore, the objective of the Pathways to Cardiovascular Disease Prevention and Impact of Specialty Referral in Under-Represented Racial/Ethnic Minorities with HIV (PATHWAYS) study was to determine the likelihood of, and factors associated with, a cardiologist encounter among UREG PWH with borderline CVD risk. Given the observed underutilization of cardiologists for CVD care among women within the general population, our analysis also focused on sex-stratified risk determinants and encounters with specialists.

## Methods

The PATHWAYS study (NCT04025125, https://clinicaltrials.gov/ct2/show/NCT04025125) is a multi-institutional collaborative observational study assessing the frequency of cardiology encounters for UREG PWH with borderline risk of atherosclerotic CVD (ASCVD) in the Southern U.S. Additionally, the study evaluates the multilevel (patient and clinical environment level) [21, 22] determinants associated with specialist encounters.

### Patient population

We utilized patient-level data from the electronic health records (EHRs) collected retrospectively from selected institutions in the Stakeholders, Technology, and Research (STAR) Clinical Research Network (CRN) that had been harmonized into the National Patient-Centered Clinical Research Network (PCORnet) Common Data Model (CDM) [23]. The PCORnet CDM provides a standardized representation of common EHR data domains and is coupled with a data curation process to assess data quality. The institutions included Duke University (Durham, North Carolina), Vanderbilt University (Nashville, Tennessee), Medical University of South Carolina (Charleston, South Carolina), and Wake Forest Baptist Health (Winston-Salem, North Carolina).

**Fig. 1:**
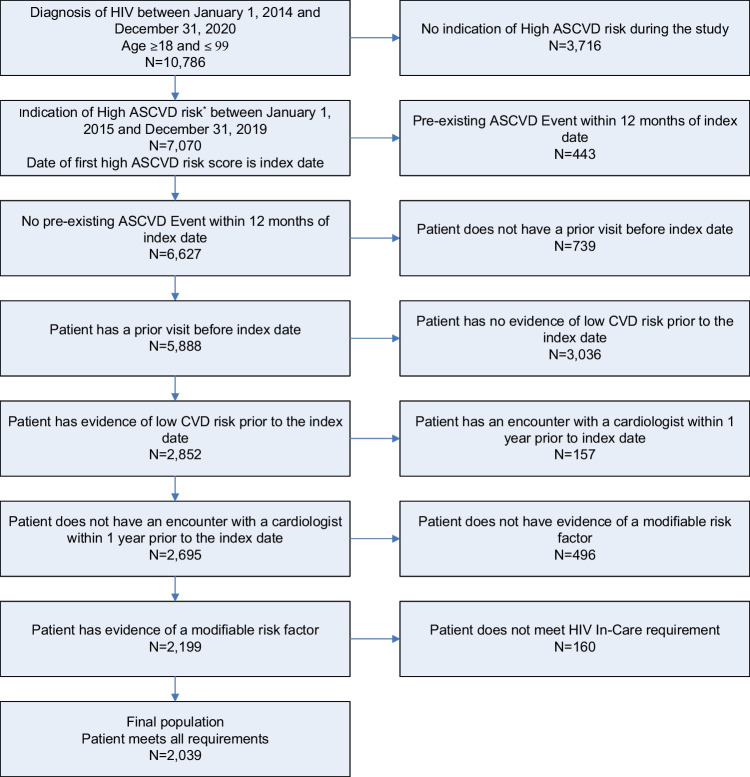
Consort Diagram of the PATHWAYS Cohort. During the eligibility period (2014–2020), inclusion and exclusion criteria for HIV status, race and clinical contact were applied. We began the observation period in 2015 to allow for a 1-year lookback period to observe data needed to calculate ASCVD risk scores. The observation period ended in 2019 to allow for at least 1 year of observation through the end of 2020. Pre-existing ASCVD events included acute myocardial infarction, heart failure, acute coronary syndrome, stable or unstable angina, arterial revascularization (including coronary arterial or peripheral), stroke, transient ischemic attack, or atherosclerotic peripheral arterial disease based on ICD-9/10 codes. ASCVD, atherosclerotic cardiovascular disease

Figure 1 outlines how we applied the eligibility, inclusion, and exclusion criteria. Participants considered for inclusion were UREG patients retained in HIV care (defined by an HIV viral load laboratory test, a prescription for antiretroviral therapy [ART], and/or an encounter with an HIV provider in the 12 months prior to their index date) [1] at these institutions between January 1, 2014 and December 31, 2020. We defined UREGs as patients who self-reported as Black/African American, American Indian or Alaska Native, Asian, Multiple Race, or Hispanic who had confirmed HIV infection with any clinical contact at a participating site. We did not restrict our definition of UREG beyond race and ethnicity. We additionally restricted our analysis to persons with newly elevated ASCVD risk between January 1, 2015 and June 30, 2019 defined as: (1) age ≥ 40 years and a 10-year ASCVD risk ≥ 5% (calculated by the pooled cohort equation [PCE]) or ≥ 7.5% (calculated by the simplified Framingham risk score) [24–26], or (2) age < 40 years and a lifetime ASCVD risk ≥ 39%. We chose these thresholds to define a low to borderline risk group [27]. We defined the patient’s index date as the first date of elevated ASCVD risk, and we observed all cardiology encounters through December 31, 2020. Follow-up was censored if a patient died during the follow-up period without a cardiologist visit; the patient was followed to the end of the study (December 31, 2020) without a cardiology visit; or the patient’s last ambulatory visit occurred prior to the end of the study (censored at 6 months after the last follow-up).

We then excluded patients who had an encounter with a cardiologist or an ASCVD event in the 12 months prior to their index date. Additionally, we excluded patients if they lacked ≥ 1 modifiable risk factor (ie, current smoker status, body mass index [BMI] > 25 kg/m^2^, or a diagnosis of diabetes mellitus, hypertension, or dyslipidemia as defined in the next section) assessed in the 12 months prior to their index date.

### Variable definitions

The primary outcome for this analysis was time from index date to first outpatient encounter with a cardiology specialist. Data on initiation of a referral to a cardiologist were not available. The health care provider associated with a visit was classified based on the provider specialty listed in the CDM and the provider’s National Provider Identifier restricted to ambulatory clinic visits (see Supplementary Table 1). Encounters for cardiac diagnostic testing alone were not included as such testing could be ordered by any clinician. Baseline comorbidities were defined based on diagnoses coded on the index date or within 2 years prior. A full list of all diagnoses and codes applied for this study are in Supplementary Table 2. Blood pressure control was defined as the most recent systolic blood pressure < 140 mmHg and diastolic blood pressure < 90 mmHg among those with hypertension. Lipid control was defined as non-HDL cholesterol < 130 mg/dL among those with dyslipidemia. Baseline laboratory measures were based on the closest measurement from 2 years prior through 6 months after the index date to maximize data available to calculate CVD risk. Baseline medications were based on relevant prescriptions on the index date or within 13 months prior to account for prescriptions potentially written annually. Supplementary Table 3 contains the full list of medication concepts and drug names. Health insurance was determined from payers listed on the index encounter or within 13 months prior to the index encounter to maximize recorded insurance from infrequent clinic visits, and a patient could have multiple insurance types assigned (ie, Medicare/Medicaid, private, Ryan White HIV/AIDS Program [RWHAP], other, and none). The Charlson Comorbidity Index was determined using the method of Glasheen et al. [28]*.* Baseline viral suppression was defined as an HIV viral load < 200 copies/mL or undetectable. A patient was classified as having a history of AIDS if they had a CD4 + T-cell count < 200 cells/uL at any time within 2 years prior to through 6 months after the index date or had diagnoses associated with AIDS [28]. Clinical encounter visits separated by ≥ 7 days were considered unique episodes of care.

### Statistical analysis

We described demographic, social, and clinical characteristics in the overall cohort and by sex. Continuous variables are described using their median and interquartile range (IQR). Categorical variables are described by their frequency (out of available sample size if missing values) and percentages. Comparisons between categories were performed with a Kruskal–Wallis test for continuous variables and a chi-squared test for categorical variables.

We calculated the cumulative percentage of PWH with a cardiology visit after their first elevation in ASCVD risk. Cox proportional hazards regression was used to characterize the univariable association of patient demographics, HIV status, medical history, CVD risk factors, and health care utilization with time to first cardiology visit. Hazard ratios with 95% confidence intervals (CIs) and p-values are provided for each univariable test.

To identify the subset of variables most strongly associated with time to first cardiology visit, we used multivariable Cox proportional hazards analysis with backward variable selection and p > 0.05 as the exit criteria. Baseline variables considered as candidates for inclusion in the model were identified a priori and included age, sex, insurance type, rural vs. urban residence, social deprivation index score, CD4 + T-cell count, viral suppression, ART prescription, atrial fibrillation, Charlson comorbidity score, estimated glomerular filtration rate (eGFR), BMI categories, blood pressure control (not hypertensive, hypertensive controlled, hypertensive not controlled), lipid control (not diagnosed hyperlipidemia, controlled hyperlipidemia, uncontrolled hyperlipidemia), current smoker status, and primary provider (HIV vs. primary care). Observations with missing values were excluded from descriptive and univariable analyses. For the multivariable modeling, multiple imputation (25 imputed datasets) was used to fill in missing covariate values (missingness summarized by sex in **Supplementary Table 4**). Models were run on each imputed dataset, and estimates were obtained by aggregating results across imputations. All p-values are two-sided, and a value < 0.05 is considered statistically significant. No adjustment was made for multiple comparisons. SAS V9.4 (SAS Institute, Cary, North Carolina) was used to perform the statistical analyses.

### Institutional ethics review and approval

The Duke University Health System Institutional Review Board (IRB) served as the single IRB of record (Pro00101663, Pro00101104) with approval of a waiver of informed consent. Using a SMART IRB agreement, all sites relied on Duke for IRB review and approval. All procedures followed were in accordance with the ethical standards of the Declaration of Helsinki of the World Medical Association.

## Results

We identified 2039 individuals (median age: 45 years [IQR: 36–50], 52% female, 94% Black or African American) across the four institutions (Table 1). Women were on average 6 years older and had slightly greater BMI, systolic blood pressure, and diastolic blood pressure than men (all p < 0.001, Table 1). The median CD4 + T-cell count was 605 cells/uL (IQR: 378–858), and most participants (89%) had achieved viral suppression. ART use in the 2 years surrounding the date when eligibility was met was overall similar between the groups.

**Table 1:** Demographic, Social, and Clinical Characteristics at Baseline by Sex

Characteristic^a^	Overall (N = 2039)	Female (N = 1061)	Male (N = 978)	P-value
Demographics				
Age, yrs	45.0 (36.0, 50.0)	48.0 (41.0, 53.0)	42.0 (32.0, 47.0)	< .001
Race, n/N (%)				< .001
American Indian or Alaska Native	9/1875 (0.5)	3/1012 (0.3)	6/863 (0.7)	
Asian	35/1875 (1.9)	5/1012 (0.5)	30/863 (3.5)	
Black or African American	1755/1875 (93.6)	979/1012 (96.7)	776/863 (89.9)	
White	65/1875 (3.5)	18/1012 (1.8)	47/863 (5.4)	
Multiple Race	11/1875 (0.6)	7/1012 (0.7)	4/863 (0.5)	
Hispanic ethnicity, n/N (%)	225/2035 (11.1)	68/1060 (6.4)	157/975 (16.1)	< .001
Vital measures				
BMI, kg/m2 [N]	29.0 (25.1, 34.1) [1927]	31.5 (26.4, 37.2) [1013]	27.3 (24.2, 30.9) [914]	< .001
Systolic BP, mmHg [N]	135.0 (124.0, 144.0) [2039]	139.0 (128.0, 148.0) [1061]	131.5 (120.0, 140.0) [978]	< .001
Diastolic BP, mmHg [N]	81.0 (75.0, 90.0) [2039]	82.0 (76.0, 90.0) [1061]	80.0 (74.0, 88.0) [978]	< .001
Other relevant medical history				
Atrial fibrillation, n (%)	28 (1.4)	15 (1.4)	13 (1.3)	0.870
Hepatitis C, n (%)	172 (8.4)	112 (10.6)	60 (6.1)	< .001
Charlson Comorbidity Index, score [N]^b^	4.0 (3.0, 6.0) [2039]	4.0 (3.0, 6.0) [1061]	3.0 (3.0, 6.0) [978]	0.024
eGFR, mL/min/1.73m2 [N]^c^	88.4 (72.7, 102.9) [1953]	86.6 (69.2, 101.5) [1001]	90.5 (76.7, 105.0) [952]	< .001
HIV characteristics				
AIDS diagnosis, n/N (%)	635/2039 (31.1)	342/1061 (32.2)	293/978 (30.0)	0.268
CD4 count, cells/uL [N]	604.5 (378.0, 858.0) [1842]	656.5 (400.0, 930.0) [930]	561.0 (360.0, 779.5) [912]	< .001
Viral suppression (< 200 copies/mL or not detected), n/N (%)	1304/1464 (89.1)	635/712 (89.2)	669/752 (89.0)	0.891
On antiretroviral therapy, n (%)	1669 (81.9)	831 (78.3)	838 (85.7)	< .001
Behavioral and social factors				
Medicare/Medicaid, n/N (%)^d^	836/2039 (41.0)	595/1061 (56.1)	241/978 (24.6)	< .001
Private insurance, n/N (%)	928/2039 (45.5)	379/1061 (35.7)	549/978 (56.1)	< .001
RWHAP insurance, n/N (%)	528/2039 (25.9)	188/1061 (17.7)	340/978 (34.8)	< .001
Other insurance, n/N (%)	96/2039 (4.7)	39/1061 (3.7)	57/978 (5.8)	0.022
No or missing insurance, n/N (%)	1181/2039 (57.9)	601/1061 (56.6)	580/978 (59.3)	0.224
Urban residence (vs. rural), n (%)^e^	1832 (89.8)	948 (89.3)	884 (90.4)	0.438
Social Deprivation Index^e^	76.0 (56.0, 89.0)	76.0 (60.0, 89.0)	75.0 (54.0, 86.0)	< .001
CVD risk factors				
Diabetes mellitus, n (%)	186 (9.1)	151 (14.2)	35 (3.6)	< .001
Hypertension, n (%)	768 (37.7)	525 (49.5)	243 (24.8)	< .001
Elevated total cholesterol, n/N (%)	452/1651 (27.4)	250/814 (30.7)	202/837 (24.1)	0.003
Elevated LDL cholesterol, n/N (%)	306/1597 (19.2)	162/791 (20.5)	144/806 (17.9)	0.184
Current smoker, n (%)	591 (29.0)	349 (32.9)	242 (24.7)	< .001
Obese (BMI > = 30 kg/m^2^), n(%)	841 (43.6)	576 (56.9)	265 (29.0)	< .001
Cardiovascular risk scores				
ASCVD risk score, % [N]	5.1 (3.6, 6.8) [1001]	4.8 (3.2, 6.9) [577]	5.3 (4.2, 6.6) [424]	
ASCVD > = 5%, n/N (%)	528/1001 (52.7)	272/577 (47.1)	256/424 (60.4)	
Framingham risk score, % [N]^f^	9.3 (8.1, 11.9) [1315]	9.3 (8.1, 11.6) [790]	9.3 (8.1, 12.3) [525]	
Framingham > = 7.5%, n/N (%)	1277/1315 (97.1)	760/790 (96.2)	517/525 (98.5)	
Lifetime risk score, % [N]	45.5 (39.1, 45.5) [650]	39.1 (39.1, 39.1) [237]	45.5 (45.5, 50.4) [413]	
Lifetime risk > = 39%, n/N (%)	650/650 (100.0)	237/237 (100.0)	413/413 (100.0)	
Laboratory measures				
Total cholesterol, mg/dL [N]	176.0 (151.0, 203.0) [1651]	181.0 (156.0, 207.0) [814]	172.0 (148.0, 200.0) [837]	< .001
HDL cholesterol, mg/dL [N]	47.0 (38.0, 58.0) [1650]	50.0 (41.0, 61.0) [813]	43.0 (36.0, 54.0) [837]	< .001
LDL cholesterol, mg/dL [N]	100.0 (81.0, 123.0) [1597]	102.0 (83.0, 125.0) [791]	98.0 (80.0, 120.0) [806]	0.013
Triglycerides, mg/dL [N]	115.0 (81.0, 171.0) [1605]	115.0 (83.0, 160.0) [793]	115.0 (81.0, 184.5) [812]	0.267
Hemoglobin, g/dL [N]	13.4 (12.1, 14.6) [1736]	12.4 (11.4, 13.4) [866]	14.4 (13.4, 15.4) [870]	< .001
Hemoglobin A1c, % [N]	5.5 (5.2, 5.9) [807]	5.6 (5.2, 6.0) [409]	5.5 (5.2, 5.7) [398]	< .001
BP control at baseline [5], n (%)	967 (47.4)	439 (41.4)	528 (54.0)	< .001
Lipid control at baseline, n (%)^g^	889 (53.9)	420 (51.7)	469 (56.0)	0.075

ASCVD, atherosclerotic cardiovascular disease; BMI, body mass index; BP, blood pressure; eGFR, estimated glomerular filtration rate; HDL, high-density lipoprotein; LDL, low-density lipoprotein; RWHAP, Ryan White HIV/AIDS Assistance Program

^a^Continuous variables are listed with the median (IQR). Categorical variables are listed as the frequency (percentage)

^b^Components are: age, myocardial infarction, congestive heart failure, peripheral vascular disease, stroke/transient ischemic attack, dementia, chronic obstructive pulmonary disease, connective tissue disease, peptic ulcer disease, liver disease, diabetes, hemiplegia, moderate to severe chronic kidney disease, solid tumor, leukemia, lymphoma, AIDS as in Glasheen et al. Charlson Comorbidity Index: ICD-9 Update and ICD-10 Translation. Am Health Drug Benefits 2109;12(4):188–197

^c^eGFR calculated from creatinine using 2009 version of the CKD-EPI equation without race component as in Levey et al. A new equation to estimate glomerular filtration rate. Ann Intern Med 2009;150(9):604–612

^d^Insurance information captures recorded insurance type at time of index encounter and 13 months prior. Because individuals could use different insurance types over time, the sums exceed 100%

^e^Residence type and Social Deprivation Index obtained by linking 5-digit zip code to the residence designation in the American Community Survey 2015 (https://www.census.gov/programs-surveys/acs/)

^f^Full version uses age, sex, total cholesterol, HDL cholesterol, systolic BP (SBP), BP treatment, current smoker status, and diabetes. Simplified version (not requiring cholesterols) uses age, BMI, SBP, BP treatment, current smoker status, and diabetes

^g^Blood pressure and lipid control defined according to prevailing guidelines during the study period

As Table 1 highlights, most individuals reported at least one encounter with no or missing insurance information during this period (58%). After that, Medicare/Medicaid (41%), private insurance (46%), and RWHAP (26%) were the most common insurance types. **Supplementary Table 5** shows the cross-tabulations of insurance types, with insurance and clinical variables demonstrating no meaningful differences in CVD risk scores across insurance types. Most individuals reported multiple insurance types, suggesting changes in insurance coverage throughout the study period.

Overweight/obesity (76%), hypertension (38%), current smoker status (29%), and elevated total cholesterol (27%) were the most common CVD risk factors across the study population, which were also more common among women. The prevalence of diabetes was higher among women (14% vs. 4%, p < 0.001). Data to calculate CVD risk scores were available on a subset of participants (72% for ASCVD risk score, 95% for Framingham risk score, and 100% for lifetime risk score, see Supplementary Table 5). In participants ≥ 40 years old, ASCVD risk scores were greater among men, with 60% of men and 47% of women having an ASCVD risk score ≥ 5%. A similar pattern was seen for lifetime ASCVD risk score in participants < 40 years old, where the median score was 46% for men and 39% for women (Table 1). Lipid control was similar among both sexes, but women less often had blood pressure control than men (41% vs. 54%, p < 0.001).

Healthcare utilization patterns are shown in Table 2. Most individuals (57%) had an infectious disease specialist as their only primary care provider. After meeting eligibility criteria based on CVD risk threshold, 283 (14%) individuals had an ambulatory visit with a cardiologist. Women were more likely to have seen a cardiologist than men (17% vs. 11%, p < 0.001). Among participants seeing a cardiologist during our study period, the time interval between meeting the CVD risk threshold and an encounter with a cardiologist was a median of 24 months (20 months in women vs. 32 months for men). Through 60 months of follow-up, the cumulative percentage of participants who were seen by a cardiologist was higher among women than men (Supplementary Fig. 1). By the end of 60 months of follow-up, less than 25% of all individuals had a cardiology visit. The most common cardiovascular diagnoses associated with these visits were related to hypertension, chest pain, other potential cardiac symptoms, and hyperlipidemia (see Supplementary Table 6).

**Table 2:** Health Care Utilization Patterns Prior to and After Index Date

Characteristic^a^	Overall (N = 2039)	Female (N = 1061)	Male (N = 978)	P-value
Visits prior to index date				
Primary provider, n (%)				< .001
HIV specialist	1171 (57.4)	558 (52.6)	613 (62.7)	
Primary care specialist	745 (36.5)	436 (41.1)	309 (31.6)	
Seeing both types of providers equally	69 (3.4)	39 (3.7)	30 (3.1)	
Unable to determine	54 (2.6)	28 (2.6)	26 (2.7)	
Number of AV visits in last 12 months [N]	5.0 (3.0, 8.0) [2023]	5.0 (3.0, 9.0) [1050]	5.0 (3.0, 7.0) [973]	< .001
Average days between clinic visits in past 12 months [N]	49.6 (31.3, 73.0) [2023]	47.6 (29.8, 72.0) [1050]	51.1 (32.9, 76.3) [973]	0.029
ED encounter in past 12 months, n (%)	507 (24.9)	309 (29.1)	198 (20.2)	< .001
Hospitalization in past 12 months, n (%)	281 (13.8)	171 (16.1)	110 (11.2)	0.001
Visits after index date				
Patients with a cardiology AV encounter, n (%)	283 (13.9)	179 (16.9)	104 (10.6)	< .001
Time to first cardiology encounter, days [N]	728.0 (365.0, 1350.0) [283]	617.0 (333.0, 1102.0) [179]	959.5 (512.0, 1613.5) [104]	

AV, ambulatory care visits; ED, emergency department

^a^Continuous variables are listed with the median (IQR). Categorical variables are listed as the frequency (percentage)

We next modeled the likelihood of an encounter with a cardiologist using both univariable and multivariable analyses. On univariable testing, older age was associated with a higher likelihood of seeing a cardiologist (HR: 1.48, 95% CI: 1.30–1.69), whereas male sex and rural residence were associated with a lower likelihood (HR: 0.60, 95% CI: 0.47–0.77; HR: 0.53, 95% CI: 0.31–0.89, respectively) (Table 3). Individuals who recently reported private, RWHAP, or other insurance types were almost 50% less likely to have an encounter with a cardiologist. Atrial fibrillation (HR: 5.32, 95% CI: 3.02–9.29), hypertension (HR: 1.59, 95% CI: 1.26–2.01), and current smoker status (HR: 1.36, 95% CI: 1.07–1.74) were among the strongest univariable predictors of an encounter with a cardiologist. Having an HIV specialist as a primary care provider (versus not) was associated with a greater likelihood of a cardiology encounter (HR: 1.63, 95% CI: 1.29–2.06), as was a greater number of ambulatory clinic visits per year (Table 3).

**Table 3:** Univariable Analysis of Predictors of an Encounter with a Cardiologist

	Raw encounter rates		Univariable analysis	
Characteristic	With characteristic (event/group)	Without characteristic (event/group)	Hazard ratio (95% CI)	*P*-value
Demographics and behavioral and social factors				
Age (per 10 years)			1.48 (1.30—1.69)	< .001
Sex, male	104/978 (10.6)	179/1061 (16.9)	0.60 (0.47—0.77)	< .001
Insurance				
Medicare/Medicaid	151/836 (18.1)	132/1203 (11.0)	1.60 (1.27—2.02)	< .001
Private insurance	103/928 (11.1)	180/1111 (16.2)	0.65 (0.51—0.83)	< .001
RWHAP insurance	48/528 (9.1)	235/1511 (15.6)	0.56 (0.41—0.76)	< .001
Other insurance	6/96 (6.3)	277/1943 (14.3)	0.57 (0.25—1.27)	0.169
No insurance or missing	205/1181 (17.4)	78/858 (9.1)	1.98 (1.52—2.57)	< .001
Rural residence	15/207 (7.2)	268/1832 (14.6)	0.53 (0.31—0.89)	0.016
SDI score (per 10 unit increase)			1.02 (0.96—1.07)	0.542
HIV care characteristics				
CD4 count (per 10 cells/mm^3)			1.00 (0.99—1.00)	0.106
Viral suppression^b^	215/1304 (16.5)	17/160 (10.6)	1.33 (0.82—2.15)	0.254
On antiretroviral therapy	236/1669 (14.1)	47/370 (12.7)	1.05 (0.77—1.44)	0.748
Other relevant medical history				
Atrial fibrillation	13/28 (46.4)	270/2011 (13.4)	5.32 (3.05—9.29)	< .001
Hepatitis C	29/172 (16.9)	254/1867 (13.6)	1.18 (0.80—1.73)	0.409
Charlson Comorbidity Index			1.13 (1.08—1.19)	< .001
eGFR, mL/min/1.73m2 (per 10 unit decrease)			1.10 (1.05—1.15)	< .001
Modifiable CVD risk factors at baseline				
BMI (per 10 unit increase)			1.02 (0.87—1.19)	0.807
Diabetes mellitus	34/186 (18.3)	249/1853 (13.4)	1.30 (0.91—1.87)	0.147
Hypertension	146/768 (19.0)	137/1271 (10.8)	1.59 (1.26—2.01)	< .001
Uncontrolled blood pressure, > = 160 mm/Hg SBP or > = 100 mm/Hg DBP among hypertensive patients				
Not hypertensive	Reference	137/1271 (10.8)		
Controlled hypertensive	117/622 (18.8)		1.57 (1.23—2.02)	< .001
Uncontrolled hypertensive	29/146 (19.9)		1.68 (1.12—2.51)	0.011
Hyperlipidemia	82/463 (17.7)	201/1576 (12.8)	1.23 (0.95—1.60)	0.110
Uncontrolled lipids, LDL cholesterol > = 160 among patients with hyperlipidemia				
Not dyslipidemia	Reference	201/1576 (12.8)		
Controlled dyslipidemia	77/432 (17.8)		1.24 (0.95—1.62)	0.111
Uncontrolled dyslipidemia	5/31 (16.1)		1.14 (0.50—2.59)	0.751
Current smoker	104/591 (17.6)	179/1448 (12.4)	1.36 (1.07—1.74)	0.012
Health care utilization				
Primary provider is an HIV specialist	141/814 (17.3)	139/1171 (11.9)	1.63 (1.29—2.06)	< .001
AV visits in past 12 months				
1 to 5 AV visits	Reference	113/1091 (10.4)		
6 to 10 AV visits	105/638 (16.5)		1.54 (1.18—2.00)	0.002
11 or more AV visits	62/294 (21.1)		2.03 (1.49—2.77)	< .001

AV, ambulatory clinic visit; BMI, body mass index; eGFR, estimated glomerular filtration rate; RWHAP, Ryan White HIV/AIDS Assistance Program; SDI, social deprivation index

^a^Event is defined as an encounter with a cardiologist

^b^Defined as an HIV RNA level < 200 copies/mL

All variables included for selection in our multivariable modeling are shown in Table 4. Patients with a diagnosis of atrial fibrillation were over 3 times more likely to see a cardiologist (HR: 3.20, 95% CI: 1.81–5.66) in multivariate testing. Having at least one recent encounter with no or missing insurance was associated with twice the likelihood of seeing a cardiologist (HR: 2.11, 95% CI: 1.61–2.76). Older patients and those with a higher BMI and more comorbidities were also more likely to have an encounter with a cardiologist.

**Table 4:** Association Between Patient Characteristics and Cardiologist Visit

	Multivariable Analysis^a^	
Characteristic^b^	Hazard ratio (95% CI)	*P*-value
Age (per 10 years)	1.46 (1.27—1.68)	< .001
Insurance		
Medicare/Medicaid Insurance	1.12 (0.84—1.50)	0.436
Private insurance	0.84 (0.63—1.12)	0.241
RWHAP	0.62 (0.45—0.87)	0.005
Other insurance	0.60 (0.26—1.37)	0.224
No insurance	2.11 (1.61—2.76)	< .001
Rural vs. urban residence	0.57 (0.34—0.96)	0.034
Atrial fibrillation	3.20 (1.81—5.66)	< .001
BMI (per 10 unit increase)	1.18 (1.01—1.38)	0.039
Charlson Comorbidity Index	1.12 (1.06—1.18)	< .001
Current smoker	1.27 (0.98—1.64)	0.069

BMI, body mass index

^a^Backward variable selection using p > .05 as exit

^b^Selection variables include: age, sex, insurance type (Medicare/Medicaid, private, Ryan White HIV/AIDS Program (RWHAP), other, and none), rural vs. urban, SDI score, CD4 count, viral suppression, ART prescription, atrial fibrillation, Charlson comorbidity score, eGFR, BMI categories, blood pressure control (not hypertensive, hypertensive controlled, hypertensive not controlled), lipid control (not diagnosed hyperlipidemia, controlled hyperlipidemia, uncontrolled hyperlipidemia), current smoker status, and primary provider (HIV vs. primary care)

## Discussion

Using real-world data, we examined the relationship between multidimensional factors and encounters with a cardiology specialist in a cohort of UREG PWH with borderline risk of ASCVD in the Southern U.S. In this cohort, we found that: (1) a small proportion of patients had subsequent encounters with a cardiologist after crossing ASCVD risk thresholds, and (2) the strongest determinants of an encounter with a cardiologist were related to a diagnosis of CVD, insurance type, and urban residence.

Our study is the first, to our knowledge, to report on the frequency of cardiology encounters for UREG PWH at borderline CVD risk. Access to specialty care is usually driven by referrals from a primary care physician. Most patients had an infectious disease specialist as their primary care provider, which was associated with a greater likelihood of seeing a cardiologist than having a primary care physician. This may be related to the reported discomfort among infectious disease specialists with managing CVD [29, 30]. In the HIV Outpatient Study, for example, less than 50% of hypertensive PWH being seen at HIV specialty clinics were treated according to prevailing guidelines [31]. Similar to the current analysis, men and those with private insurance were less likely to meet treatment guidelines. Okeke et al. also demonstrated worse compliance with hypertension treatment guidelines when comparing PWH treated in HIV clinics to primary care offices [32]. Our findings, however, may suggest a shift in care patterns in response to the growing attention to CVD risk management in PWH. While the effect of clinician specialty was attenuated in multivariate modeling, more research is needed to understand the relevant provider and patient factors related to more effective linkage to specialty care [29].

Patient and provider characteristics have repeatedly been shown to impact care for CVD. We are aware of no other published studies that have addressed the expected frequency of cardiology encounters for this borderline risk group with HIV. Prior work related to the pattern of cardiology referrals for individuals without manifest CVD has focused on care patterns for individuals with non-specific chest pain in primary care clinics or in non-U.S. health care systems [16, 33–35]. Cook et al. showed that women had less access to cardiologists than men despite a similar burden of CVD [16]. In a German cohort of primary care practices, for example, 14.5% of patients eventually had a cardiology encounter over a period of 1 year [33]. Our study is unique in its focus on UREG PWH and on individuals without known CVD. Given the doubling of cardiovascular events in PWH compared with rates estimated by current cardiovascular disease risk calculators, we suggest that a higher rate than observed in our study (13.9%) may be reasonable clinically [36]. While overall rates of encounters with cardiology are unexpectedly low, we found that UREG women had more and earlier visits with a cardiologist after meeting our ASCVD risk threshold than men. Sex differences were attenuated in the multivariate analysis perhaps due to the large difference in age between men and women in this cohort.

Health insurance is a critical component of access to appropriate healthcare in the U.S. for PWH [37]. In the current analysis, reporting “no insurance” on at least one recent encounter was strongly linked to a subsequent encounter with a cardiologist. While this may seem counterintuitive on the surface, the nuances of how healthcare is covered for PWH offers some potential explanations. The RWHAP is the “payer of last resort” for PWH who meet state-dependent income thresholds to cover costs related to HIV care and other medical comorbidities [38]. RWHAP fee schedules and formularies differ based on state and local policies [39]. Due to the constraints imposed by a lack of traditional health insurance, HIV clinic staff must often find innovative ways to assist under- or uninsured patients. For example, McManus et al. highlighted that when one state’s RWHAP program ended its coverage for CVD risk factor management, clinic staff were instrumental in finding alternative sources of coverage and CVD risk factor care was preserved despite the apparent lack of insurance coverage [40]. Webel et al. have also shown that sponsor-supported services substantially shape preventative CVD care in HIV clinics in North Carolina and Ohio [41].

Individual institutions also vary greatly in how coverage for services are recorded in the EHR. Specifically, patients classified as having “no insurance” at our participating sites could either be: (1) truly uninsured with limited access to providers due to cost constraints, (2) uninsured but met income criteria for institutional charitable programs that waived out-of-pocket expenses for basic access to subspecialty providers and encounters, or (3) in a state where RWHAP covered subspecialty services, but administratively the EHR did not recognize such coverage as insurance. We posit that the strong linkage we observed between reporting no insurance and seeing a cardiologist is likely due to institutional efforts to link patients to specialty care using non-traditional methods, HIV clinic reorganization to manage patient’s comorbid medical conditions, and innovative support programs, similar to previous studies [42, 43].

The strengths of this analysis include a study population from a real-world setting enriched for UREG PWH. Several limitations are also worth noting. The scope of data was largely limited to elements currently collected via the CDM, which largely represents information captured in structured fields within the EHR, although we did also use complementary stand-alone approaches to extract elements, such as encounters with specialists. Data on initiation of a referral to a cardiologist were not available. We chose the point at which an individual’s CVD risk crossed a particular threshold and linked this time point to future visits with a cardiologist. Because it is possible that provider’s awareness of crossing this threshold may have been later in time, we opted for a long follow-up period to observe future cardiology visits. We excluded individuals with prior ASCVD events, which limits generalizability and may introduce detection bias as it relates to the prevalence estimates of risk factors. Exposures, such as insurance type or others, are susceptible to misclassification due to the nature of these EHR data. In addition, only clinical data related to encounters within these health systems were included, and we may have missed encounters with primary care providers or cardiologists outside of these systems, such as in community-based clinics. Lastly, in the absence of a consensus on risk score thresholds for cardiology referrals among PWH, we used elevated and borderline risk thresholds based on guidelines for the general population [27].

In summary, in UREG PWH with borderline CVD risk, clinical, geographic, and socio-economic factors are associated with subsequent encounters with a cardiologist. These factors represent potential directed targets to increase use of cardiology specialty care for UREG PWH. The relationship between cardiology specialty care and subsequent CVD events in this population warrants further research.

### Supplementary Information

Below is the link to the electronic supplementary material.

Supplementary file1 (DOCX 80 KB)

## Data Availability

The data that support the findings of this study are available from the corresponding author upon reasonable request and will be made publicly available through the National Institutes of Health.
