# Supplementary material for: Cardiology Encounters for Underrepresented Racial and Ethnic Groups with Human Immunodeficiency Virus and Borderline Cardiovascular Disease Risk
Source: J Racial Ethn Health Disparities. 2023 May 9;11(3):1509–19. doi: 10.1007/s40615-023-01627-0 (PMC10632543; doi:10.1007/s40615-023-01627-0)

**SUPPLEMENTARY INFORMATION**

**Cardiology Encounters for Underrepresented Racial and Ethnic Groups with Human Immunodeficiency Virus and Borderline Cardiovascular Disease Risk**

Gerald S. Bloomfield, MD, MPH^1,2^; C. Larry Hill, PhD^2^; Karen Chiswell, PhD^2^; Linda Cooper^3^; Shamea Gray, MDV^1^; Chris T. Longenecker, MD^4^; Darcy Louzao, PhD^2^; Keith Marsolo, PhD^2,5^; Eric G. Meissner, MD, PhD^3^; Caryn G. Morse, MD, MPH^6^; Charles Muiruri, PhD^5^; Kevin L. Thomas, MD^1,2^; Eric J. Velazquez, MD^7^; Joseph Vicini, MDiv^8^; April C. Pettit, MD, MPH^8^; Gretchen Sanders^2^; and Nwora Lance Okeke, MD, MPH^1,5^

^1^Department of Medicine, Duke University School of Medicine, Durham, North Carolina, USA

^2^Duke Clinical Research Institute, Duke University School of Medicine, Durham, North Carolina, USA

^3^Department of Medicine, Medical University of South Carolina, Charleston, South Carolina, USA

^4^Division of Cardiology and Department of Global Health, University of Washington, Seattle, Washington, USA

^5^Department of Population Health Sciences, Duke University School of Medicine, Durham, North Carolina, USA

^6^Wake Forest University Health Sciences, Winston-Salem, North Carolina, USA

^7^Section of Cardiovascular Medicine, Yale University, New Haven, Connecticut, USA

^8^Department of Medicine, Vanderbilt University, Nashville, Tennessee, USA

**Address for Correspondence:**

Gerald S. Bloomfield, MD, MPH

Duke Clinical Research Institute

300 W. Morgan Street

Durham, NC 27701

Phone: 919-668-8702

Fax: 919-668-7032

Email: [gerald.bloomfield@duke.edu](mailto:gerald.bloomfield@duke.edu)

**Supplementary Table 1.** Provider Specialty Taxonomy Codes

| **Description** | **Taxonomy code** |
| --- | --- |
| **Cardiologist** |  |
| Internal Medicine Advanced Heart Failure and Transplant Cardiology | 207RA0001X |
| Internal Medicine Cardiovascular Disease | 207RC0000X |
| Internal Medicine Interventional Cardiology | 207RI0011X |
| Nuclear Medicine Nuclear Cardiology | 207UN0901X |
| Pediatrics Pediatric Cardiology | 2080P0202X |
| Thoracic Surgery (Cardiothoracic Vascular Surgery) | 208G00000X |
| Physical Therapist Cardiopulmonary | 2251C2600X |
| Technician, Cardiology | 246W00000X |
| Spec/Tech, Cardiovascular | 246X00000X |
| Spec/Tech, Cardiovascular Invasive Specialist | 246XC2901X |
| Spec/Tech, Cardiovascular Vascular Specialist | 246XC2903X |
| Spec/Tech, Cardiovascular Sonography | 246XS1301X |
| Radiologic Technologist Cardiovascular-Interventional Technology | 2471C1101X |
| **HIV specialist** |  |
| Allergy & Immunology | 207K00000X |
| Internal Medicine Infectious Disease | 207RI0200X |
| Physician Assistant | 363A00000X |
| Nurse Practitioner | 363L00000X |
| **Primary care specialist** |  |
| Family Medicine | 207Q00000X |
| Internal Medicine | 207R00000X |
| Physician Assistant Medical | 363AM0700X |
| Nurse Practitioner Adult Health | 363LA2200X |
| Nurse Practitioner Family | 363LF0000X |
| Nurse Practitioner Gerontology | 363LG0600X |
| Nurse Practitioner Primary Care | 363LP2300X |

Provider specialties were identified through two main processes. To identify cardiologists, a list of specialty taxonomy codes was obtained from the Medicare and Medicaid (CMS) datasets (<https://taxonomy.nucc.org/>). Review of the taxonomy codes by the Pathways steering committee identified those codes related to a cardiology specialty. Designations for advanced practice providers specific to cardiology were not present during the time period of the study. The second process was to identify the primary provider type (HIV primary provider or primary care provider) for each patient. To identify potential provider specialties, a list of specialties where a patient had at least 3 ambulatory visits in the past 2 years was obtained from the PCORNet CDM data. The list was reviewed by the Pathways steering committee to identify specialties associated with primary care or care by an HIV specialist.

**Supplementary Table 2.** Diagnosis Concepts and International Classification of Disease (ICD) Codes for Comorbidities

| **Diagnosis concept** | **ICD-9 codes** | **ICD-10 codes** |
| --- | --- | --- |
| Alcohol abuse | 291.x, 303.x – 305.03, 357.5, 425.5, 535.3x, 571.0 – 571.3, 760.71, 980.x | F10.x – F10.99, G62.1, I42.6, K29.2x, K70.x |
| Atrial fibrillation/flutter | 427.3x, 427.4x, 427.8 – 427.9 | I48.x – I49.9 |
| Cerebrovascular disease | 430 – 438.9, 346.6x, 362.3x | G45 – G46, H34.x, I60.x – I69.998, G43.6x, H34.1x, R29.70x – R29.742 |
| Chronic pulmonary disease | 416.x, 490 – 496, 500 – 508.8 | I27.8, I27.9, J40.x – J45.x, J47.x, J60.x – J68.4, J70.x |
| Cocaine use | 304.2 – 305.63, 760.75, 970.81 | F14 – F14.99, P04.41, R78.2, T40.5x |
| Coronary artery bypass graft | 414.xx | Z95.5 |
| Coronary artery disease | 411.x – 414.9, V45.8x | I20.x, I24.x, I125.x, Z95.1, Z98.61, I67.2 |
| Critical limb ischemia | 440.2x | I70.2xx |
| Dementia | 290 – 294.1, 331.2 | F01 – F03.91, G30 – G31.1 |
| Diabetes^a^ | 249 – 250.93 | E08.x – E13.9, O24.01x – O24.03, 024.111 – O24.13 |
| Heart failure | 398.x, 402.x, 404.x, 425.x, 428.x | I09.x, I11.0, I13.x, I25.x, I42.x-I43, I50.x, P29.0 |
| Hemiplegia or paraplegia | 334.1 – 344.9 | G04.1, G11.4, G80.1 – G83.9 |
| Hepatitis C | 070.41, 070.44, 070.51, 070.54, 070.7 – 070.71, V02.62 | B17.1x, B18.2, B19.2x |
| HIV | 042, 079.53, V08 | B20, B97.35, O98.7x – O98.73, Z21 |
| Hyperlipidemia^a^ | 272 – 272.4 | E78.0 – E78.5 |
| Hypertension^a^ | 362.11, 401.x – 405.99, 437.2, 642.x – 642.94 | H35.031 – H35.039, I10 – I13.2, I15 – I16.9, I67.4, O10.0x – O11.9 |
| Malignancy | 140.x - 165.x, 170.x – 172.x, 174.x – 176.x, 179 – 195.x, 200.x – 208.x, 238.6 | C00.x – C26.x, C30.x – C34.x, C37.x – C41.x, C43.x, C45.x – C58.x, C60.x – C76.x, C81.x – C85.x, C88.x, C90.x – C96.x |
| Metastatic solid tumor | I96 – I99.2 | C77 – C80.2 |
| Liver disease | O70.xx, 570 – 573.x, V42.7, 456.x, 572.x | B18.x, I85.0, I86.4, K70.x – K76.7, Z94.4 |
| Myocardial infarction | 410.x, 411.x – 411.81, 412, 429.79 | I21.x, I22.x, I23.7, I24.0, I25.x |
| Obesity | 278 – 278.03, V85.3 – V85.45 | E66.x, Z68.3 – Z68.45 |
| Peptic ulcer disease | 531 – 534.91 | K25 – K28.9 |
| Percutaneous coronary intervention | V45.82 | Z95.5, Z98.61 |
| Peripheral vascular disease | 250.7 – 250.73,O93.0, 437.3, 414.0 – 414.4, 437.0, 440.0 – 440.32, 440.4, 440.8, 440.9, 441.x, 443.1 – 443.9, 444.22 – 444.89, 445.02, 447.1, 459.9, 707.1x, 785.4, V43.4, 557.x, | E08.5x – E13.59, I25.1x – I25.89, I67.2, I70.x – I79.8, I96, L97 – L97.929 |
| Renal disease | 403.01 – 404.93, 582.x – 588.0, V42.0, V45.1, V56.x | I12.0, I13.1, N03.2 – N05.7, N18.x – N19, N25.0, Z49.x |
| Rheumatic disease | 446.5, 710.x, 714.x, 725 | M05.x – M06.9, M32.x – M36.0 |
| Stable angina | 411, 413.0, 413.9, 429.79 | I20, I20.1 – I20.9, I23.7 |
| Tobacco use | 305.1, 649.0 – 649.04, 989.84 | F17, F17.2x, O99.33x, T65.2, T65.2 – T65.22x, T65.29 - T65.294x |
| Unstable angina | 411 – 411.1 | I20.0 |

^a^Diabetes mellitus was further defined by use of anti-diabetic medication. Hypertension was further defined by a systolic blood pressure >140 mmHg or a diastolic blood pressure > 90 mmHg, and dyslipidemia was further defined by a total cholesterol >200 mg/dL, LDL cholesterol >130 mg/dL, or HDL cholesterol <50 mg/dl (women) or <40 mg/dl (men) even in the absence of a diagnosis code.

**Supplementary Table 3.** Medication Concepts and Drug Names

| **Medication concept** | **Drug names** |
| --- | --- |
| ACE inhibitor | Benazepril, Captopril, Cilazapril, Enalapril, Fosinopril, Lisinopril, Moexipril, Perindopril, Quinapril, Ramipril, Trandolapril |
| Aldosterone antagonists | Canrenone, Eplerenone, Spironolactone |
| Alpha-glucosidase inhibitors | Acarbose, Miglitol |
| Angiotensin receptor neprilysin inhibitor | Sacubitril |
| Angiotensin receptor blockers | Azilsartan, Candesartan, Eprosartan, Irbesartan, Losartan, Olmesartan, Telmisartan, Valsartan |
| Anticoagulant | Apixaban, Betrixaban, Dabigatran, Dalteparin, Dicumarol, Edoxaban, Enoxaparin, Fondaparinux, Rivaroxaban, Warfarin |
| Antiplatelet | Abciximab, Aspirin, Cangrelor, Cilostazol, Clopidogrel, Dipyridamole, Eptifibatide, Prasugrel, Ticagrelor, Ticlopidine, Tirofiban, Vorapaxar |
| Beta-blocking agents | Acebutolol, Atenolol, Betaxolol, Bisoprolol, Carteolol, Carvedilol, Esmolol, Labetalol, Metoprolol, Nadolol, Nebivolol, Oxprenolol, Penbutolol, Pindolol, Propranolol, Sotalol, Timolol |
| Biguanides | Metformin |
| Dihydropyridine calcium channel blockers | Amlodipine, Clevidipine, Felodipine, Isradipine, Nicardipine, Nifedipine, Nimodipine, Nisoldipine |
| Dopamine-2 agonists | Bromocriptine |
| Dpp-4 inhibitors | Alogliptin, Linagliptin, Saxagliptin, Sitagliptin |
| Drugs used in nicotine dependence | Bupropion, Nicotine, Varenicline |
| GLP 1 agonists | Albiglutide, Dulaglutide, Exenatide, Liraglutide, Lixisenatide, Semaglutide |
| Insulins and analogues | Degludec, Detemir, Glargine, Glulisine, Insulin, Lispro |
| Integrase strand transfer inhibitors | Bictegravir, Cabotegravir, Dolutegravir, Elvitegravir, Raltegravir |
| Loop diuretics | Bumetanide, Ethacrynate, Furosemide  Torsemide |
| Meglitinides | Nateglinide, Repaglinide |
| Non-dihydropyridine calcium channel blockers | Diltiazem, Verapamil |
| Non-nucleoside reverse transcriptase inhibitors | Delavirdine, Doravirine, Efavirenz, Etravirine, Nevirapine, Rilpivirine |
| Nucleoside reverse transcriptase inhibitors | Abacavir, Didanosine, Emtricitabine, Lamivudine  Stavudine, Tenofovir, Zalcitabine, Zidovudine |
| Other antihypertensives | Chlorthalidone, Hydrochlorothiazide, Metoprolol, Metyrosine, Oxprenolol |
| Other antiretroviral therapy | Cobicistat, Enfuvirtide, Maraviroc, Fostemsavir, Ibalizumab |
| Other diuretics | Acetazolamide, Amiloride, Chlorothiazide, Chlorthalidone, Hydrochlorothiazide, Indapamide, Methyclothiazide, Metolazone, Triamterene |
| Other non-statin lipid-lowering therapies | Bezafibrate, Cholestyramine, Choline, Clofibrate, Colesevelam, Colestipol, Dextrothyroxine, Fenofibrate, Gemfibrozil, Lomitapide, Mipomersen, Policosanol, Probucol |
| Pcsk-9 inhibitors | Alirocumab, Evolocumab |
| Protease inhibitors | Amprenavir, Atazanavir, Cobicistat, Darunavir, Fosamprenavir, Indinavir, Lopinavir, Nelfinavir, Ritonavir, Saquinavir, Tipranavir |
| Selective cholesterol absorption inhibitors | Ezetimibe |
| Sglt2 inhibitors | Canagliflozin, Dapagliflozin, Empagliflozin, Ertugliflozin |
| Statin medications | Atorvastatin, Cerivastatin, Fluvastatin, Lovastatin, Pitavastatin, Pravastatin, Rosuvastatin, Simvastatin |
| Thiazolidinediones | Pioglitazone, Rosiglitazone, Troglitazone |

**Supplementary Table 4.** Missingness of Risk Factor Characteristics by Sex

|  | **Sex** | |
| --- | --- | --- |
| **Characteristic^a^** | **Female**  **(N=1061)** | **Male**  **(N=978)** |
| Age, yrs | 0 | 0 |
| Total cholesterol, mg/dL | 247 (23%) | 141 (14%) |
| HDL cholesterol, mg/dL | 248 (23%) | 141 (14%) |
| Antihypertensive treatment | 0 | 0 |
| Current smoker | 0 | 0 |
| Diabetes mellitus | 0 | 0 |
| BMI, kg/m2 | 48 (5%) | 64 (7%) |
| Systolic BP, mmHg | 0 | 0 |
| Diastolic BP, mmHg | 0 | 0 |

BMI, body mass index; BP, blood pressure.

^a^Risk factor characteristics consist of variables needed to calculate 10-year ASCVD risk, Framingham 10-year CVD risk, and lifetime risk scores.

**Supplementary Table 5.** Insurance Cross-Tabulation by Insurance Types, Sex, Age, and CVD Risk Scores

|  | **Insurance type^b^** | | | | |
| --- | --- | --- | --- | --- | --- |
| **Characteristic^a^** | **Medicare/Medicaid** | **Private** | **RWHAP** | **Other** | **None or missing** |
| **Insurance type^b^** |  |  |  |  |  |
| Medicare/Medicaid, n/N (%) | 836/836 (100.0) | 146/928 (15.7) | 69/528 (13.1) | 17/96 (17.7) | 402/1181 (34.0) |
| Private insurance, n/N (%) | 146/836 (17.5) | 928/928 (100.0) | 308/528 (58.3) | 19/96 (19.8) | 490/1181 (41.5) |
| RWHAP, n/N (%) | 69/836 (8.3) | 308/928 (33.2) | 528/528 (100.0) | 38/96 (39.6) | 362/1181 (30.7) |
| Other insurance, n/N (%) | 17/836 (2.0) | 19/928 (2.0) | 38/528 (7.2) | 96/96 (100.0) | 61/1181 (5.2) |
| No or missing insurance, n/N (%) | 402/836 (48.1) | 490/928 (52.8) | 362/528 (68.6) | 61/96 (63.5) | 1181/1181 (100.0) |
| **Clinical variables** |  |  |  |  |  |
| Female sex, n/N (%) | 595/836 (71.2) | 379/928 (40.8) | 188/528 (35.6) | 39/96 (40.6) | 601/1181 (50.9) |
| Age, yrs | 47.0 (41.0, 52.0) | 44.0 (34.0, 50.0) | 43.0 (34.0, 49.0) | 40.0 (33.0, 46.5) | 44.0 (35.0, 50.0) |
| ASCVD risk score, % [N] | 5.3 (3.6, 7.3) [447] | 5.0 (3.6, 6.6) [468] | 5.1 (3.5, 6.4) [253] | 4.4 (2.6, 7.1) [39] | 5.0 (3.4, 6.6) [593] |
| ASCVD >= 5%, n/N (%) | 252/447 (56.4) | 245/468 (52.4) | 138/253 (54.5) | 16/39 (41.0) | 297/593 (50.1) |
| Framingham risk score, % [N] | 9.6 (8.2, 12.4) [638] | 9.3 (8.1, 11.7) [540] | 9.5 (8.2, 12.2) [285] | 9.3 (8.1, 13.1) [45] | 9.3 (8.2, 11.7) [741] |
| Framingham >= 7.5%, n/N (%) | 616/638 (96.6) | 525/540 (97.2) | 280/285 (98.2) | 44/45 (97.8) | 725/741 (97.8) |
| Lifetime risk score, % [N] | 39.1 (39.1, 50.2) [169] | 45.5 (45.5, 45.5) [345] | 45.5 (45.5, 50.4) [213] | 45.5 (45.5, 45.5) [47] | 45.5 (39.1, 50.2) [411] |
| Lifetime risk >= 39%, n/N (%) | 169/169 (100.0) | 345/345 (100.0) | 213/213 (100.0) | 47/47 (100.0) | 411/411 (100.0) |

ASCVD, atherosclerotic cardiovascular disease; RWHAP, Ryan White HIV/AIDS Program.

^a^Continuous variables are listed with the median (IQR). Categorical variables are listed as the frequency (percentage).

^b^Insurance type categories are not mutually exclusive.

**Supplementary Table 6.** Diagnoses at Time of Cardiology Encounter for 283 Encounters

| **Diagnoses at cardiology encounter** | **Number (percentage of encounters)** |
| --- | --- |
| Noncardiac diagnosis | 100 (35.3) |
| Hypertension | 56 (19.8) |
| Chest pain | 53 (18.7) |
| Diagnosis not grouped | 49 (17.3) |
| Other potentially cardiac symptoms | 45 (15.9) |
| Hyperlipidemia | 28 (9.9) |
| Arrhythmia | 27 (9.5) |
| Cardiovascular risk factors | 26 (9.2) |
| Heart failure | 25 (8.8) |
| Palpitations | 17 (6.0) |
| Coronary artery disease | 16 (5.7) |
| Structural heart disease | 13 (4.6) |
| Other cardiac diseases | 10 (3.5) |
| Cardiovascular risk factors | 8 (2.8) |
| Myocardial infarction | 6 (2.1) |
| Peripheral vascular disease | 6 (2.1) |
| Preoperative evaluation | 5 (1.8) |
| Pericardial disease | 4 (1.4) |
| Abnormal cardiac imaging/testing | 2 (0.7) |
| Murmur | 1 (0.4) |

Rows are not mutually exclusive because multiple diagnoses may have been coded on the cardiology encounter. Overall, 93 (33%) cardiology encounters had no diagnoses associated with the specific encounter ID.

**Supplementary Figure 1.** Cumulative Incidence of Cardiology Visits According to Sex


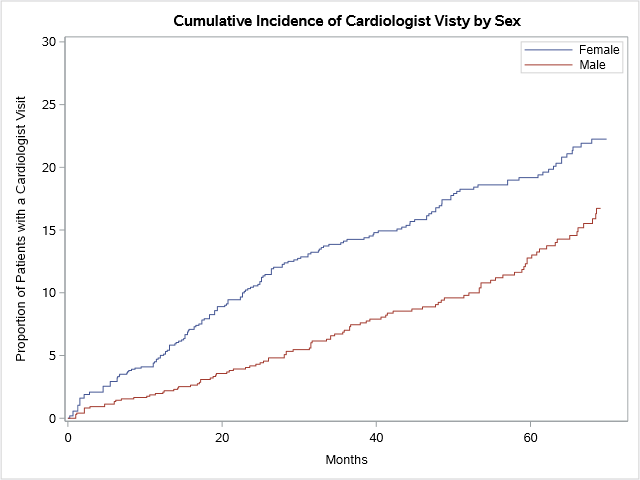

Supplement: Supplementary file 1 — Supplementary file1 (DOCX 80 KB) [file 40615_2023_1627_MOESM1_ESM.docx]
